# Management of Gingival Reactive Lesions: A Case Series

**DOI:** 10.7759/cureus.113692

**Published:** 2026-07-30

**Authors:** Shivani G, Rekha R Koduganti, Suhail Mir, Veerendranath Reddy Panthula, Swetha Manchala

**Affiliations:** 1 Department of Periodontics, Panineeya Institute of Dental Sciences and Research Centre, Hyderabad, IND

**Keywords:** curcumin gel, diode laser excision, fibroepithelial gingival hyperplasia, gingival hyperplasia, photobiomodulation, pyogenic granuloma

## Abstract

Reactive gingival lesions are benign, tumor-like proliferations that commonly involve the gingiva and arise due to chronic irritation or inflammation. The primary treatment is complete excision of the lesion, which, along with appropriate adjunctive measures, helps improve healing and reduce recurrence. This case series presents three cases of reactive gingival lesions managed by diode laser excisional biopsy with adjunctive curcumin gel and/or photobiomodulation (PBM). The diagnoses were confirmed by histopathological examination.

## Introduction

Reactive hyperplastic lesions of the gingiva comprise a diverse group of non-neoplastic proliferations that arise in response to chronic irritation, trauma, plaque retention, calculus, ill-fitting restorations, or hormonal influences. These lesions represent an exaggerated reparative response of the gingival connective tissue and are characterized by fibrovascular or inflammatory proliferation. Clinically, they often present as localized enlargements that may resemble benign or occasionally neoplastic lesions, thereby necessitating histopathological confirmation for an accurate diagnosis [1].

Among the most frequently encountered reactive gingival lesions are fibroepithelial hyperplasia (FEH), pyogenic granuloma (PG), and gingival hyperplasia (GH). FEH typically manifests as a firm, sessile, fibrous enlargement resulting from persistent low-grade irritation [2]. In contrast, PG is a highly vascular, fast-growing lesion composed of proliferating capillaries and granulation tissue, often associated with trauma, hormonal changes, or poor oral hygiene [3]. GH represents diffuse or localized gingival enlargement often linked to chronic plaque accumulation, systemic factors, or medication-induced overgrowth [4].

The introduction of diode lasers (810-980 nm) in soft-tissue periodontal surgery has revolutionized the management of oral lesions. These lasers show a strong affinity for hemoglobin and melanin, enabling precise cutting with excellent hemostasis, an important advantage when managing vascular lesions such as PG. Laser excision has been shown to reduce intraoperative bleeding, postoperative pain, surgical time, and the need for suturing while improving patient comfort [5].

Adjunctive agents and therapies have also gained attention for their role in enhancing wound healing. Curcumin, a bioactive compound derived from *Curcuma longa*, possesses anti-inflammatory, antioxidant, antimicrobial, and wound-modulating effects. It downregulates pro-inflammatory cytokines such as IL-6, IL-1β, and TNF-α while promoting fibroblast proliferation, collagen deposition, and angiogenesis [6]. Similarly, photobiomodulation (PBM), also known as low-level laser therapy (LLLT), has demonstrated beneficial effects on tissue regeneration through mitochondrial activation, ATP synthesis, increased microcirculation, and enhanced collagen remodeling. Clinical research confirms accelerated wound healing and decreased postoperative pain following PBM in periodontal surgery [7].

Objective evaluation of postoperative wound healing is crucial in clinical research. The Landry, Turnbull, and Howley healing index provides a standardized, reproducible method to assess soft-tissue healing based on color, granulation tissue, bleeding, and epithelialization, making it ideal for postoperative assessment in periodontal therapy [8]. Although diode laser excision of reactive gingival lesions has been previously reported, this case series is among the few to combine laser excision with two adjunctive therapies, namely curcumin gel and PBM, and to evaluate outcomes using a standardized healing index across three distinct reactive lesion types (FEH, PG, and GH) within a single series.

## Case presentation

Case 1: Fibroepithelial hyperplasia

A 58-year-old female patient reported to the Department of Periodontics with a chief complaint of a small, persistent growth on the gingiva in the mandibular posterior region for the past three months. The patient’s medical history was non-contributory, and no systemic or hormonal factors were identified. Oral hygiene history revealed inadequate plaque control and occasional trauma from brushing. Intraoral examination revealed a firm, sessile, nodular enlargement on the marginal gingiva interdentally between 35 and 36. The lesion measured approximately 6-7 mm in diameter. The surface appeared smooth, pale pink, and non-ulcerated, mimicking a chronic fibrous lesion. The lesion did not bleed on probing and was non-tender. Based on the clinical presentation, a provisional diagnosis of FEH was made. Differential diagnoses considered included peripheral ossifying fibroma, PG, and peripheral giant cell granuloma.

Treatment

**Figure 1:**
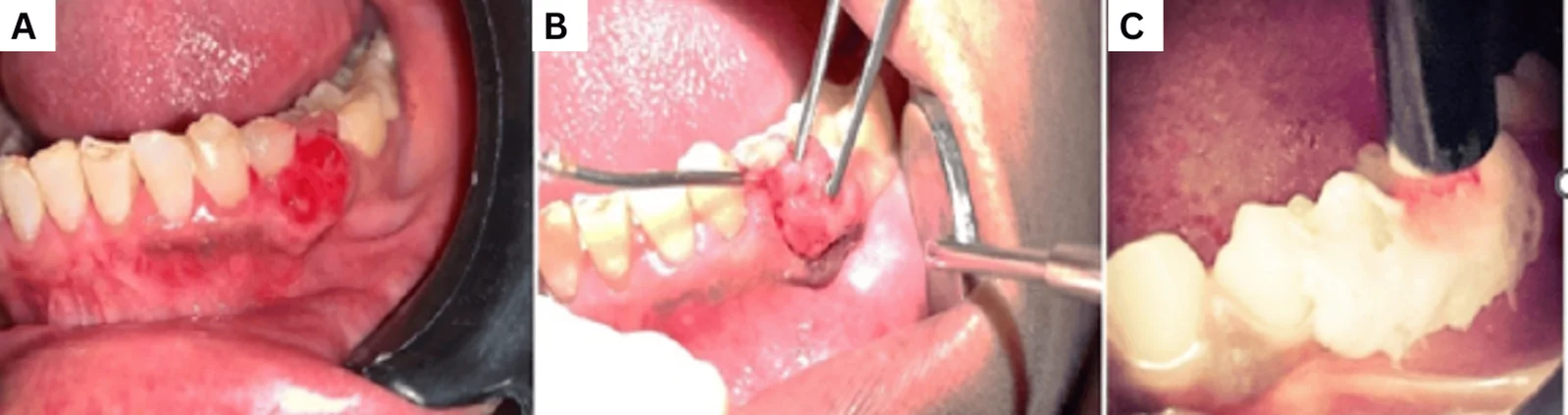
Case 1: Preoperative, intraoperative, and immediate postoperative photographs (A) Preoperative photograph, (B) excision of the lesion using a diode laser, and (C) immediate postoperative photograph showing topical application of curcumin gel and photobiomodulation.

The lesion was excised under local anesthesia using a diode laser Photon s10 operating at 980 nm with a 50 µm optical fiber in continuous-wave contact mode at 2.5 W, delivering a total energy of 6-12 J over an irradiation time of 30-60 seconds. After excision of the lesion, curcumin gel was applied, and PBM (noncontact mode at 0.5 W and 680 nm for 30-60 seconds) was performed once a week for four weeks. The procedure resulted in minimal intraoperative bleeding and did not require suturing. After three months, the surgical site healed uneventfully (Figure 1, Panels A-C).

Histopathological Report

**Figure 2:**
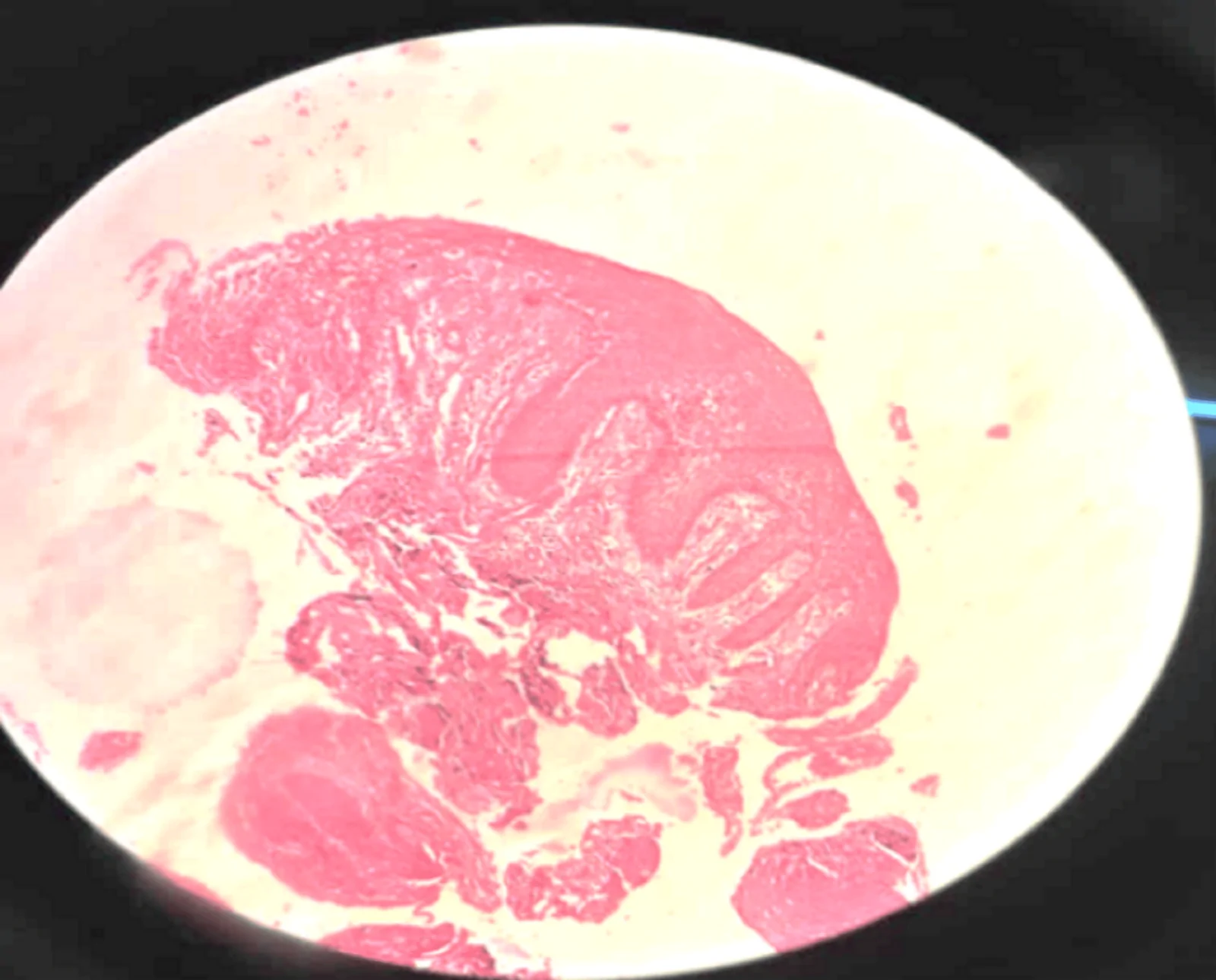
Case 1: Histopathological evaluation (hematoxylin and eosin (H&E) stained, 4x magnification)

Microscopic examination revealed para-keratinized stratified squamous epithelium overlying dense, fibrous connective tissue with minimal chronic inflammatory infiltrate. No atypical or dysplastic changes were observed. These features confirmed the diagnosis of FEH (Figure 2). 

Postoperative Healing and Follow-Up

**Figure 3:**
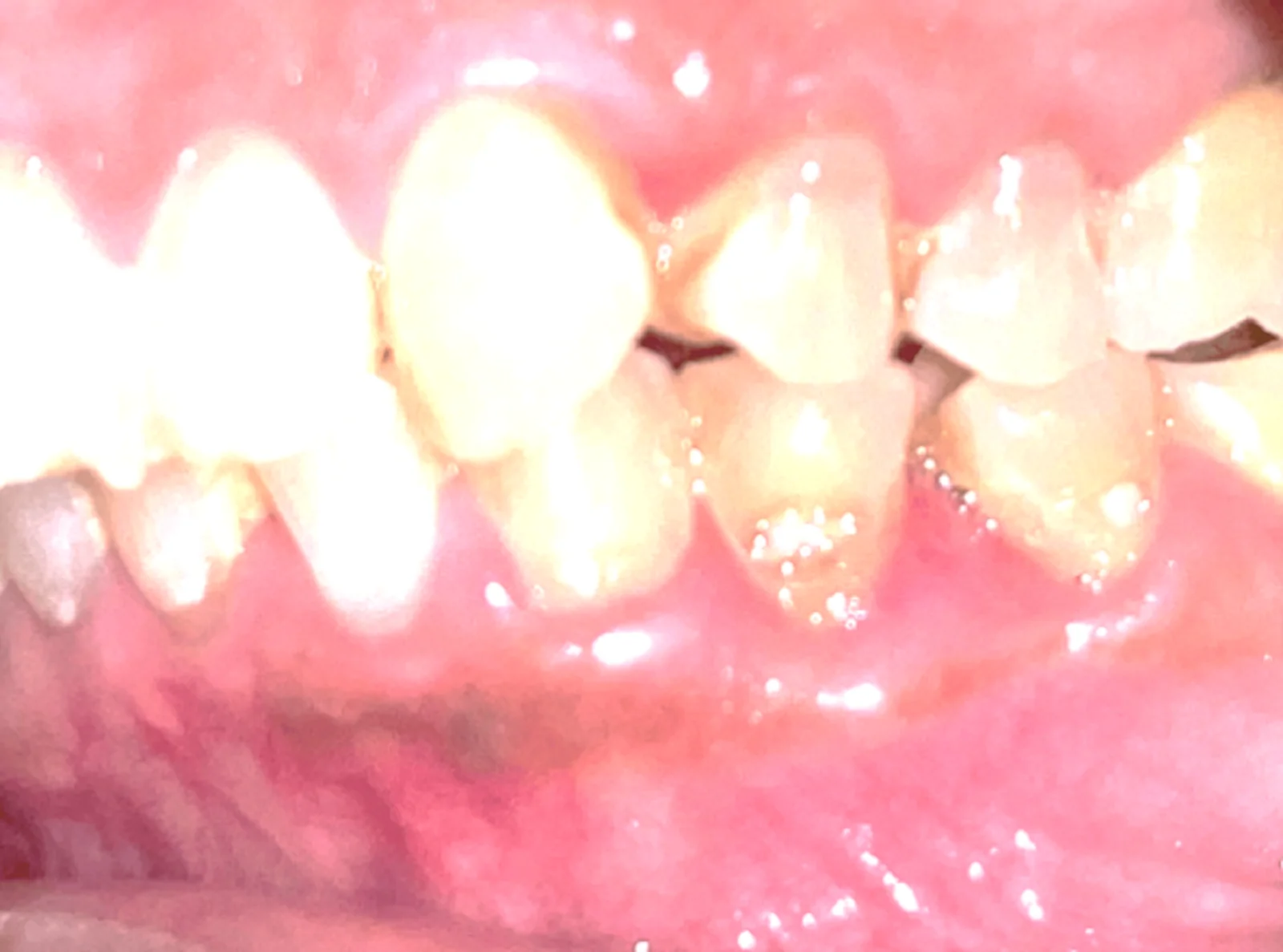
Case 1: Postoperative photograph after three months

Healing was evaluated using the Landry, Turnbull, and Howley healing index at one week, two weeks, and one month. At the end of the first week, there was mild inflammation at the surgical site, no bleeding, and initial epithelialization was observed. A score of 3 was given as per the criteria for the healing index. After two weeks, the operated site appeared pink with well-formed epithelium, and a score of 4 was given. By the end of the fourth week, there was complete epithelialization, normal contour, and no inflammation observed, and a score of 5 was given. The patient reported minimal postoperative discomfort. No complications or adverse reactions were observed. At the three-month follow-up, the surgical site displayed stable gingival architecture with no recurrence (Figure 3).

Outcome

The combination of diode laser excision, curcumin gel (commercially available as Curenext; manufactured by Akums Drugs & Pharmaceuticals Ltd, India, and marketed by Abbott India Ltd, India), which was applied topically to the surgical site once daily for four weeks, and PBM produced an excellent healing response with superior early wound maturation. This multimodal approach was effective for FEH by enhancing tissue regeneration, minimizing inflammation, and ensuring a predictable clinical outcome.

Case 2: Pyogenic granuloma

A 19-year-old female patient presented with a complaint of a rapidly growing, reddish swelling on the gingiva in the mandibular posterior region lasting for approximately six weeks. The patient reported frequent bleeding during brushing and occasionally during chewing. There was no history of systemic illness or medication use. Her oral hygiene habits were irregular, with noticeable plaque and local irritants. On intraoral examination, a soft, erythematous, lobulated, sessile swelling was observed, located on the gingiva on the buccal aspect of 36. The surrounding gingiva exhibited moderate inflammation; 36 was vital with normal probing depths. A provisional diagnosis of PG was made based on the clinical features. Differential diagnoses considered included peripheral giant cell granuloma and peripheral ossifying fibroma.

Treatment

**Figure 4:**
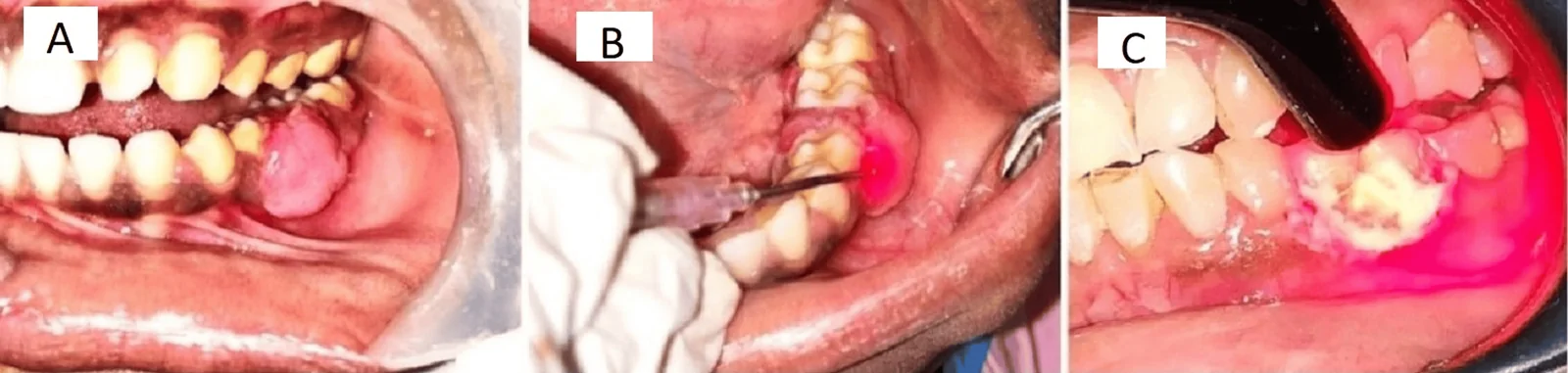
Case 2: Preoperative, intraoperative, and immediate postoperative photographs (A) Preoperative photograph, (B) excision of the lesion with diode laser, and (C) immediate postoperative photograph (photobiomodulation).

Following adequate local anesthesia, the lesion was excised using a diode laser (810-980 nm) in continuous contact mode at 1-1.5 W. Due to the lesion’s high vascularity, the diode laser provided exceptional hemostasis, ensuring a clean surgical field. No sutures were required. Before histopathological examination, the specimen was stored in 10% formalin. PBM was applied immediately after excision (noncontact mode at 0.5 W and 680 nm for 30-60 seconds) and during follow-up visits to promote wound healing and reduce inflammation. After three months, the healing was good at the surgical site (Figure 4, Panels A-C).

Histopathological Findings

**Figure 5:**
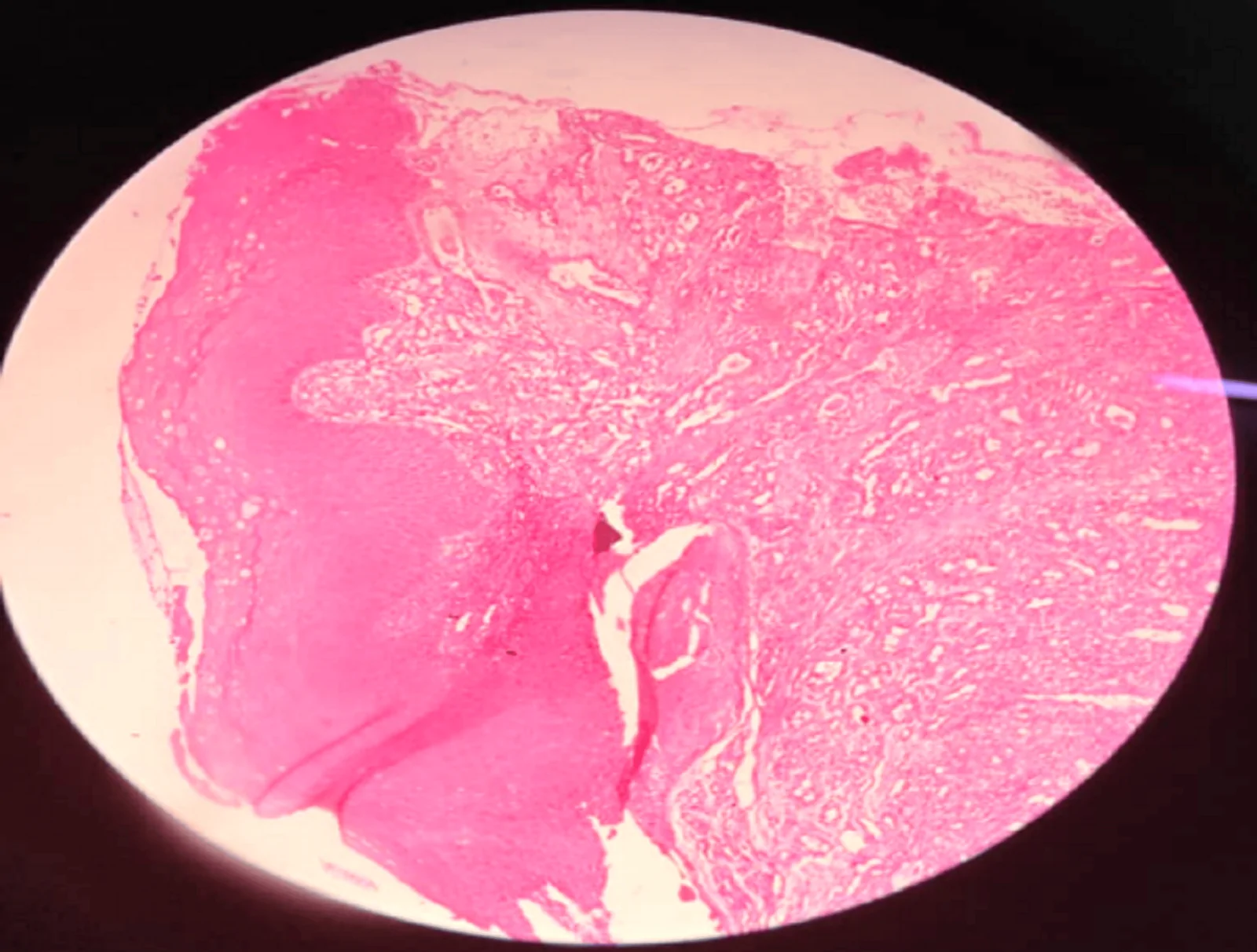
Case 2: Histopathological evaluation (hematoxylin and eosin (H&E) stained, 4x magnification)

Microscopic evaluation showed hyperplastic granulation tissue characterized by numerous capillary-sized blood vessels lined by plump endothelial cells and embedded in a loose connective tissue matrix with mixed inflammatory infiltrate. These findings confirmed the diagnosis of PG (Figure 5).

Postoperative Healing and Follow-Up

**Figure 6:**
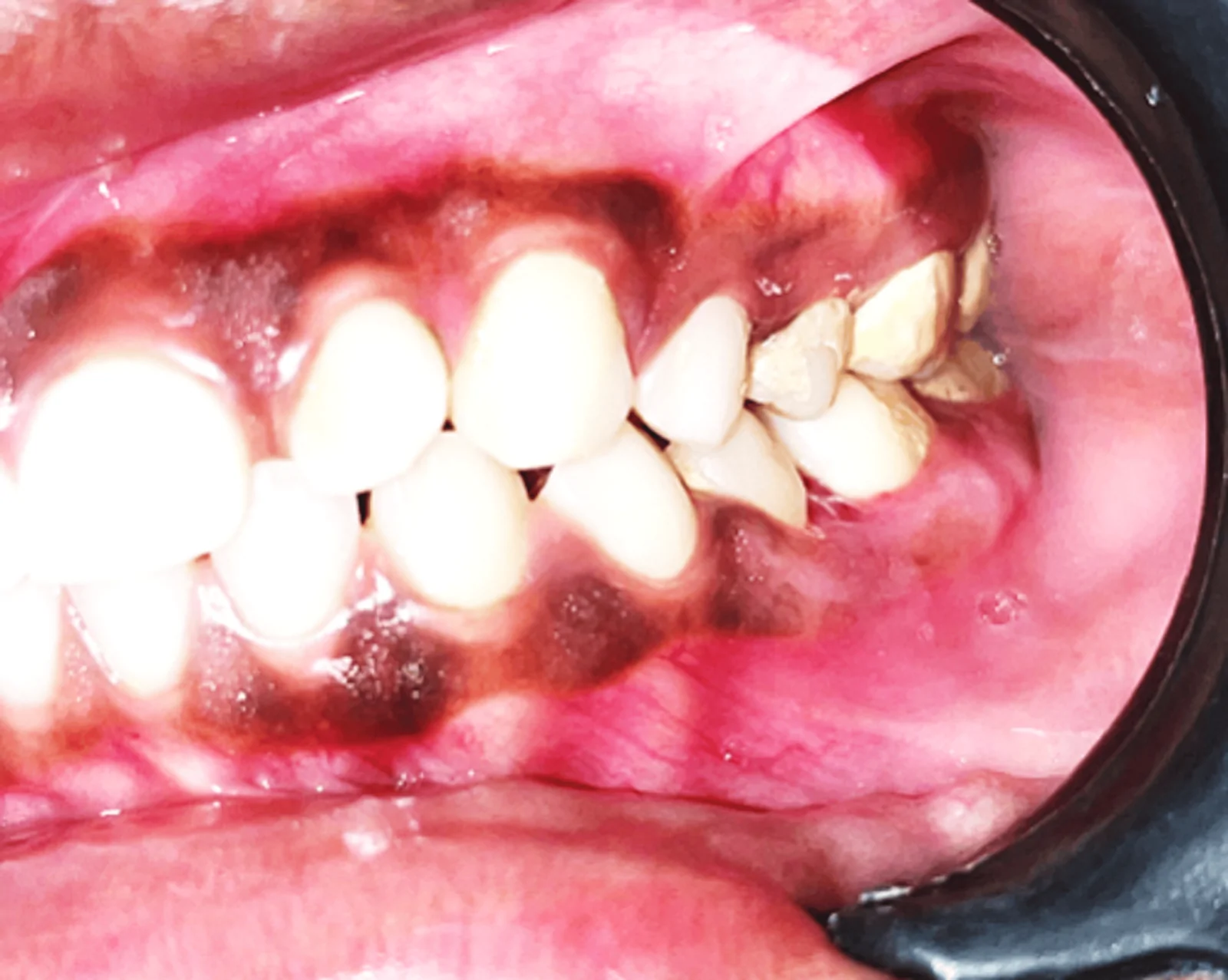
Case 2: Postoperative photograph after three months

Healing was good with minimal inflammation, initial epithelialization, and reduced bleeding within the first week; a score of 3 was given according to the healing index criteria. At the end of two weeks, the surgical site had healed better with well-formed epithelium, and a score of 4 was given. By the end of the fourth week, there was complete epithelialization, normal contour, and no inflammation observed, and a score of 5 was given. At the three-month follow-up, no recurrence was noted, confirming complete lesion resolution (Figure 6).

Outcome

The combination of diode laser excision and PBM provided a minimally invasive, bloodless procedure with rapid healing and excellent patient comfort. PBM significantly contributed to faster wound maturation, making it an essential adjunct for highly vascular lesions like PG.

Case 3: Gingival hyperplasia

A 36-year-old female patient reported to the clinic with concerns regarding swollen gums in the maxillary anterior region, which interfered with routine brushing. The swelling had been present for nearly four months and was gradually increasing in size. The patient had poor oral hygiene, with no relevant medical or drug history that could contribute to gingival enlargement. On clinical evaluation, a firm, fibrotic gingival overgrowth involving the marginal and papillary gingiva in the maxillary central and lateral region was observed. Plaque accumulation and mild gingival inflammation were noted, but periodontal probing depths were not significantly deep. A provisional diagnosis of GH (fibrous type) was made. Differential diagnoses included drug-induced gingival overgrowth and hereditary gingival fibromatosis, both considered unlikely given the absence of a contributory drug or medical history. 

Treatment

**Figure 7:**
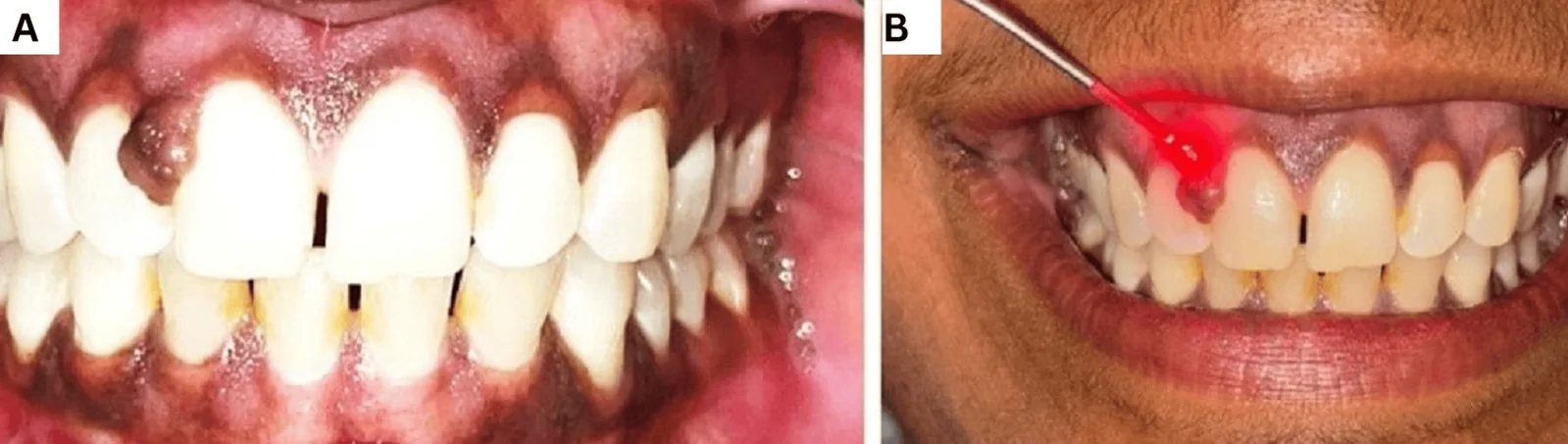
Case 3: Preoperative and intraoperative photographs (A) Preoperative photograph and (B) excision with diode laser.

A diode laser (810-980 nm) with a power setting of 1-1.5 W in contact mode was used to excise the hyperplastic tissue. The laser provided clean tissue removal with minimal bleeding. Adjunctive curcumin gel and PBM were not used in this case, as GH is a comparatively fibrous, less vascular lesion in which excision alone is sufficient for uneventful healing. The procedure was quick, comfortable, and performed without sutures. The tissue specimen was sent for histopathological examination. The patient was recalled after one week for follow-up, and at the third-month follow-up, satisfactory healing was observed (Figure 7, Panels A and B).

Histopathological Report

**Figure 8:**
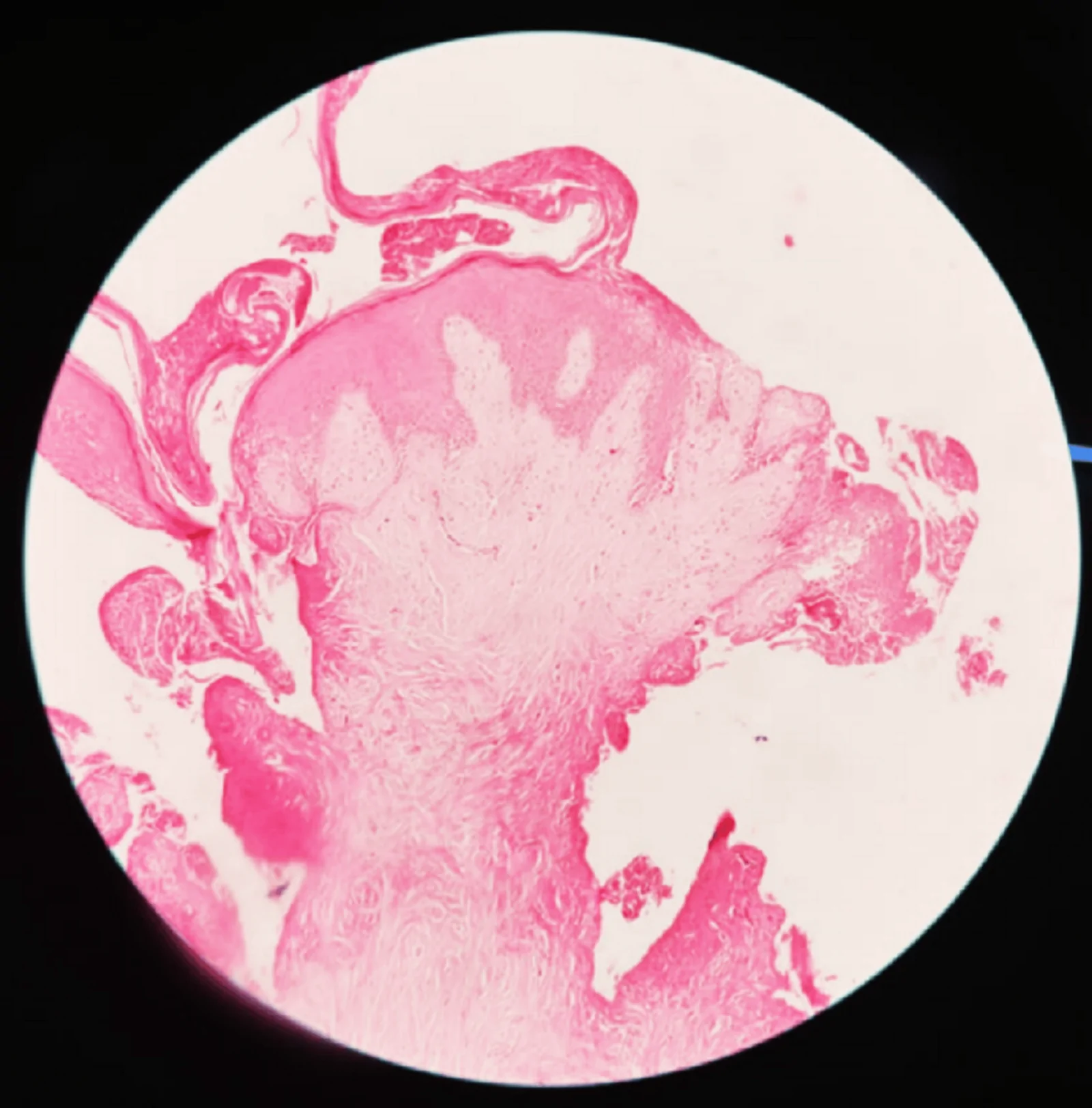
Case 3: Histopathological evaluation (hematoxylin and eosin (H&E) stained, 4x magnification)

Histology revealed dense collagenous connective tissue with elongated rete ridges and minimal inflammatory infiltrate, consistent with fibrous GH (Figure 8).

Postoperative Healing

**Figure 9:**
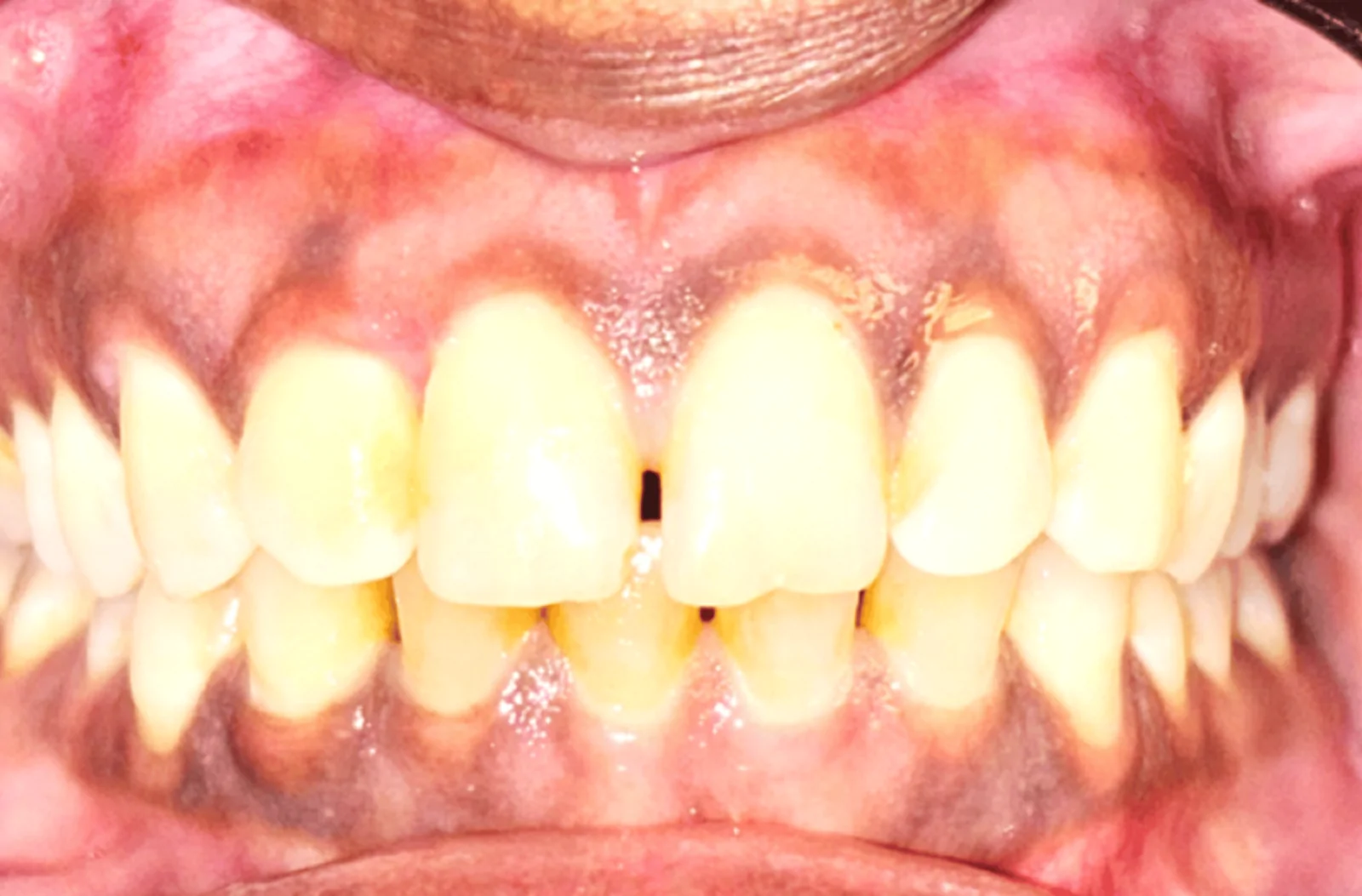
Case 3: Postoperative photograph after three months

Healing was monitored clinically. One week after excision, the tissue appeared slightly inflamed with initial epithelialization, and a score of 3 was given. By the end of three weeks, the tissue appeared healthy with epithelialization nearly complete, and a score of 3-4 was given. By the fourth week, the gingiva exhibited a firm, healthy appearance with restored contour, and a score of 4 was recorded. At three months, no recurrence or complications were observed (Figure 9).

Outcome

Laser excision alone provided a predictable and efficient treatment for GH, offering patient comfort and satisfactory functional and aesthetic outcomes. As GH is less vascular and more fibrotic, excision alone was sufficient without the need for adjunctive PBM or curcumin therapy.

## Discussion

Reactive gingival lesions such as FEH, PG, and GH arise as localized, non-neoplastic proliferations triggered by chronic irritation, plaque accumulation, hormonal influences, or trauma. Their clinical appearance often overlaps with benign and neoplastic pathologies, necessitating careful evaluation and histopathological confirmation for accurate diagnosis [1]. In the present case series, all three lesions were successfully managed with diode laser excision. However, the healing outcomes of Case 1 and Case 2, which were supported by adjunctive therapies, curcumin gel, and PBM, were appreciated more. The use of the diode laser has revolutionized oral soft-tissue surgery due to its affinity for hemoglobin and melanin, resulting in precise incisions with excellent hemostasis. This feature is particularly beneficial when treating highly vascular lesions such as PG, where scalpel surgery often results in significant bleeding. Studies consistently report reduced intraoperative bleeding, improved visibility, decreased postoperative discomfort, and faster healing following diode laser excision. Some researchers demonstrated that diode-laser excision of PG achieved superior bleeding control and faster healing than the conventional method [9]. Another study compared the conventional scalpel technique with the diode laser in the excision of PG. Twenty-one patients participated. It was observed that in the diode laser-treated group, the speed of incision, time taken, and intraoperative bleeding were much less compared to the group treated by conventional scalpel [10]. Similarly, other researchers reported low recurrence rates following diode-laser treatment of reactive gingival lesions, further confirming its clinical reliability [11]. These findings align with outcomes in the present study, where laser excision offered clean surgical fields and uneventful postoperative recovery in all cases.

PBM was used in the FEH and PG cases as an adjunct to diode laser excision. PBM enhances soft-tissue healing by stimulating mitochondrial cytochrome c oxidase, increasing ATP synthesis, accelerating collagen formation, and promoting angiogenesis. These biologic mechanisms contribute to faster wound closure and improved tissue quality. Strong evidence supports PBM’s therapeutic benefits [12]. A systematic review affirmed PBM’s effectiveness in enhancing oral wound healing and reducing postoperative inflammation [13]. The improved healing index scores in Case 1 and Case 2 further validate these findings. In periodontal applications, curcumin gel has been observed to accelerate postoperative healing, improve epithelialization, and reduce gingival inflammation [14,15]. In Case 1, curcumin gel contributed to early reduction in inflammation and improved healing index scores, supporting the existing evidence for its therapeutic efficacy. In this series, cases treated with adjunctive PBM and curcumin consistently demonstrated faster progression from “Good” to “Excellent” healing compared with excision alone, emphasizing the value of multimodal therapy. Importantly, none of the lesions in the present report recurred during follow-up. Larger prospective studies with long-term follow-up and biomarker analysis would help further establish these findings and support the development of standardized treatment protocols for reactive gingival lesions.

Limitations of adjunctive therapies

Topically applied curcumin is generally ​​​​​safe and well-tolerated. However, some patients develop mild side effects like skin irritation, itching, or contact dermatitis. Therefore, a skin patch test should be done first. PBM is a drug-free, safe, and cost-effective treatment that shows optimal clinical outcomes. No immediate adverse effects have been documented. Patients may rarely experience numbness and increased pain at the application site, typically resolving within a day. In this case series, the patients tolerated both treatment protocols well. 

## Conclusions

Diode laser excision provides a safe, efficient, and minimally invasive approach for managing reactive gingival lesions. Objective healing index scores further validate the benefit of multimodal therapy. Personalized treatment planning based on lesion characteristics ensures optimal outcomes and reduces recurrence. Adjunctive therapies were associated with favorable healing outcomes. However, larger controlled studies are needed to confirm these findings. 
